# EphA4 Activation of c-Abl Mediates Synaptic Loss and LTP Blockade Caused by Amyloid-β Oligomers

**DOI:** 10.1371/journal.pone.0092309

**Published:** 2014-03-21

**Authors:** Lina M. Vargas, Nancy Leal, Lisbell D. Estrada, Adrian González, Felipe Serrano, Katherine Araya, Katia Gysling, Nibaldo C. Inestrosa, Elena B. Pasquale, Alejandra R. Alvarez

**Affiliations:** 1 Departamento de Biología Celular y Molecular, Laboratorio de Señalización Celular, Facultad de Ciencias Biológicas, P. Universidad Católica de Chile, Santiago, Chile; 2 Departamento de Biología Celular y Molecular, Millenium Nucleus in Stress and Addiction, Facultad de Ciencias Biológicas, P. Universidad Católica de Chile, Santiago, Chile; 3 Departamento de Biología Celular y Molecular, Centro de Envejecimiento y Regeneración (CARE), Facultad de Ciencias Biológicas, P. Universidad Católica de Chile, Santiago, Chile; 4 Sanford-Burnham Medical Research Institute, La Jolla, California, United States of America; University of Leipzig, Germany

## Abstract

The early stages of Alzheimer's disease are characterised by impaired synaptic plasticity and synapse loss. Here, we show that amyloid-β oligomers (AβOs) activate the c-Abl kinase in dendritic spines of cultured hippocampal neurons and that c-Abl kinase activity is required for AβOs-induced synaptic loss. We also show that the EphA4 receptor tyrosine kinase is upstream of c-Abl activation by AβOs. EphA4 tyrosine phosphorylation (activation) is increased in cultured neurons and synaptoneurosomes exposed to AβOs, and in Alzheimer-transgenic mice brain. We do not detect c-Abl activation in EphA4-knockout neurons exposed to AβOs. More interestingly, we demonstrate EphA4/c-Abl activation is a key-signalling event that mediates the synaptic damage induced by AβOs. According to this results, the EphA4 antagonistic peptide KYL and c-Abl inhibitor STI prevented i) dendritic spine reduction, ii) the blocking of LTP induction and iii) neuronal apoptosis caused by AβOs. Moreover, EphA4-/- neurons or sh-EphA4-transfected neurons showed reduced synaptotoxicity by AβOs. Our results are consistent with EphA4 being a novel receptor that mediates synaptic damage induced by AβOs. EphA4/c-Abl signalling could be a relevant pathway involved in the early cognitive decline observed in Alzheimer's disease patients.

## Introduction

Alzheimer's disease (AD) is characterised by progressive cognitive impairment, memory loss and dementia [1]. The cognitive impairment in AD patients correlates strongly with the loss of synaptic density in the hippocampus and neocortex, accompanied by amyloid-β (Aβ) peptide accumulation [2]. The emerging view is that amyloid-β oligomers (AβOs) are a central pathological factor in early neurodegenerative events [3]. The AβO-induced changes that underlie cognitive impairment may involve the activation of signalling pathways that mediate major changes in synaptic structure and neuronal cytoskeleton organisation [4]. c-Abl is a member of the Abl family of non-receptor tyrosine kinases, which also includes Arg [5]. In addition to its function in neuronal development, c-Abl is required for the proper functioning of differentiated neurons. It has important roles in neuronal cytoskeleton remodelling, and several studies have discovered synaptic functions for c-Abl [71]. In the CA1 area of the hippocampus, c-Abl is localised in both the pre- and post-synaptic regions [6], [7], [8], and electrophysiological studies have shown that c-Abl is required for the efficient release of neurotransmitters [6]. Our laboratory demonstrated that c-Abl is especially concentrated in dendritic spines and that regulates synaptic structure and function [8].

Compelling evidence has shown that aberrant c-Abl activation participates in AD-associated neurodegeneration [9], [10], [11], [12], [13]. c-Abl is constitutively activated in AD transgenic mice, and the chronic administration of STI (STI571), a c-Abl inhibitor, significantly improves memory and learning [10], [11]. In addition, constitutively active c-Abl in the mouse forebrain induces neuronal loss and increases tau tyrosine phosphorylation in the hippocampus [13]. In the AD brain, c-Abl is detected in neurofibrillary tangles [14] and phosphorylates tau both directly [15] and through the activation of the serine-threonine kinase Cdk5 [11]. These findings support the idea that c-Abl participates in the pathogenesis of AD and other neurodegenerative diseases [9], [16], [62]. However, whether c-Abl has a role in the signalling events that mediate synapse loss induced by AβOs has not yet been evaluated. AβOs bind to synaptic structures through certain specific receptors [17], [18]; however, their role is not fully understood, and additional AβO receptors that may contribute to synaptic loss and disease pathogenesis remain to be identified [18]. Interestingly, it has been reported that members of the Eph family of receptor tyrosine kinases can interact with c-Abl and that c-Abl mediates some of the downstream effects of Eph receptors [19], [20]. The Eph receptors and their ephrin ligands perform important functions in cell-to-cell communication and are crucial in the development and proper functioning of the nervous system [21]. Recent studies have reported that AβOs bind to EphB2, leading to receptor endocytosis and degradation [22], [23], [24]. On the other hand, EphB2 overexpression has been shown to reverse the synaptic damage observed in hAPP transgenic mice [24].

Analysis of synaptoneurosomes from AD patients revealed a ∼2-fold increase in EphA4 mRNA, suggesting that this receptor may play a role in synaptotoxicity [25]. Among the Eph receptors, EphA4 is highly expressed in the nervous system, where it controls axon guidance during development and dendritic spine morphology in the adult hippocampus [26]. EphA4 has been identified as a substrate of γ-secretase, and this processing is enhanced by synaptic activity [27], [28], [29]. In addition, EphA4 activation by the ephrin-A3 ligand in hippocampal slices promotes dendritic spine retraction and pruning [27], [28]. The downstream signalling pathways that participate in EphA4-induced spine retraction involve integrin inhibition and PLC_γ_ and Cdk5 activation [27], [30]. Cdk5 activation induces phosphorylation and activation of the Rho exchange factor Ephexin1, which modulates actin cytoskeletal dynamics, leading to spine retraction [27]. It was also reported that EphA7 promotes apoptosis in neuronal precursors when bound to the ephrin-A ligand, and that overstimulation of other EphA receptors by ephrin-A ligands causes neural cell apoptosis [31]. Thus, EphA4 has been implicated in synaptic changes; however, whether AβOs modulate this signalling pathway remains to be determined.

Here, we show that AβOs induce EphA4 relocalisation and activation leading to c-Abl kinase activation. We also demonstrate that inhibition of the EphA4/c-Abl pathway prevents dendritic spine loss, the blockage of LTP induction and the apoptotic process caused by AβOs. Our results support the notion that EphA4/c-Abl signalling contributes to the development of early cognitive impairments in Alzheimer's disease patients.

## Methods

### 2.1 Ethics statement

All procedures were reviewed and approved by the Institutional Animal Care and Bioethics and Biosafety Committee of the Biological Sciences Faculty, P. Universidad Católica de Chile, which follows the Guide for the Care and Use of Laboratory Animals published by NIH.

### 2.2. Primary hippocampal cell cultures

The hippocampi were dissected from Sprague–Dawley rats at embryonic day 18 and *EphA4*-knockout and wild-type mice (*EphA4^-/-^, EphA4^+/+^*; we used *EphA4*-knockout and wild-type mice to evaluate dendritic spines and c-Abl activation in response to AβOs in s 4 A, B and 5C), and primary hippocampal cultures were prepared [9], [32]. All animals were anesthetized with CO_2_ and subsequently euthanized by cervical dislocation

Hippocampal cells were seeded onto poly-L-lysine-coated plates [32]. Cultures were maintained at 37°C in 5% CO_2_ with neurobasal growth medium (Invitrogen, Carlsbad, CA, USA) supplemented with B27 (Invitrogen, Carlsbad, CA, USA), 2 mM L-glutamine, 100 U/ml penicillin, and 100 μg/ml streptomycin (Invitrogen, Carlsbad, CA, USA). On day 2, cultured neurons were treated with 2 μM AraC for 24 h to prevent glial cell proliferation.

Hippocampal neurons were treated with either AβO or AβO-FITC (3 or 5 μM, as described in the respective figures) alone, or they were co-treated with the KYL peptide (KYLPYWPVLSSL; 30–60 μM, GenScript, Piscataway, NJ, USA Inc.) [28], [33], [34] or STI (1–5 μM, Novartis, Basel, Switzerland) [9]. The expression vector encoding Flag-EphA4 was a kind gift from Drs. Fumitoshi Irie and Yu Yamaguchi (Sanford-Burnham Medical Research Institute, USA).

### 2.3. Aβ oligomers preparation

Human synthetic Aβ1–42 (Genemed Biotechnologies Inc., San Francisco, CA, USA) was suspended in 1,1,1,3,3,3 hexafluoro-2-propanol (Sigma-Aldrich, St. Louis, MO, USA). The peptide samples were vortexed to obtain a homogeneous solution, aliquoted into microfuge tubes and lyophilised. The Aβ1–42 peptide aliquots were resuspended to 200 μM in nanopure water and vortexed briefly. Aggregation was allowed to proceed for 12 h at 4°C following the protocols by Sokolov *et al*., 2006 [35] and Arimon *et al*., 2005 [36]. To form fluorescent AβOs (AβOs-FITC), synthetic Aβ1–42 coupled to FITC (Bachem, Torrance, CA, USA) was resuspended in nanopure water (200 μM), and aggregation was allowed to proceed for 12 h at 4°C. Aβ-fibrils used in Fig. S1C were prepared in accordance with Alvarez *et al.*, 2004 [9].

### 2.4. Fluorescence labelling and quantification of dendritic spines

Hippocampal neurons were seeded onto poly-L-lysine-coated coverslips in 24-well culture plates at a density of 2.5×10^4^ cells per well; the cells were prepared according to Banker and co-workers [32]. For transfection assays, we used primary cultures of hippocampal neurons at15 DIV. These neurons were transfected using Magnetofection technology (OZ BIOSCIENCES, Marseille cedex, FRANCE) and the reagent Neuromag specific for neurons with the following short hairpin RNA plasmids: sh-EphA4; sh-c-Abl; and sh-scramble (c-Abl, sc-270357-SH; EphA4, sc-39936-SH; Santa Cruz Biotechnology, Santa Cruz, CA, USA); a GFP expression plasmid (pEGFP-C1) was used as control. At 21 DIV, the cells were treated with AβOs for 5 hours as described above. The cells were rinsed twice in ice-cold PBS, fixed with 4% paraformaldehyde 4% sucrose in PBS for 20 minutes, and permeabilized for 10 minutes with 0.2% Triton X-100 in PBS. Subsequent washes were performed with ice-cold PBS. The cells were incubated in 3% bovine serum albumin in PBS (blocking solution) for 30 min at room temperature, followed by overnight incubation at 4°C with primary antibodies. For the quantification of dendritic spines, we used TRITC–phalloidin (ECM Biosciences, Versailles, KY, USA) to label actin, which allowed us to distinguish spine morphology. The dendrites were identified according to the method described by Banker and co-workers [32]. The antibodies used for immunofluorescence were mouse anti-c-Abl, (24-11, sc-23), Santa Cruz Biotechnology, Santa Cruz, CA, USA), mouse anti-EphA4 receptor (Zymed Laboratories/Life Technologies Corporation, Carlsbad, CA, USA), rabbit anti-phospho-c-Abl (C5240, Sigma-Aldrich, St. Louis, MO, USA), and mouse anti-PSD95 (75-028, NeuroMab, Davis, CA, USA). The cells were mounted in mounting medium and visualised using a Zeiss LSM 5 Pascal confocal microscope (NA 1.4) for the acquisition of sequential images of each fluorophore at maximum resolution (1024×1024 pixels). The images were processed using ImageJ (NIH, Bethesda, MA, USA). The colocalisation between proteins was analysed using the plugin “intensity correlation analysis” and the co-localisation coefficient of Manders (M1 and M2).

### 2.5. Immunoprecipitation and immunoblotting analyses

Hippocampal neurons were plated at a density of 1.5×10^5^ cells/cm^2^ and at 15 DIV treated with AβOs. Then, the cells were washed and lysed in radioimmunoprecipitation assay (RIPA) buffer (50 mM Tris, 150 mM NaCl, 1 mM EGTA, 1 mM EDTA, 0.5% deoxycholate, 1% NP-40, and 0.1% SDS) supplemented with protease and phosphatases inhibitors (1 mM PMSF, 1 μg/ml aprotinin, 10 μg/ml leupeptin, 1 mM Na3VO4, and 50 mM NaF). The cell lysates were centrifuged at 14.000 rpm for 15 minutes at 4°C. Protein quantification was performed using the Pierce BCA Protein Assay Kit (Thermo Scientific, Waltham, MA, USA). For immunoprecipitation assays, 300–500 μg of total lysates were incubated with 2 μg of c-Abl (c-Abl (24-11) sc-23, Santa Cruz Biotechnology, Santa Cruz, CA, USA) or EphA4 (34-7900, Zymed Laboratories/Invitrogen for immunodetection, Carlsbad, CA, USA) antibodies overnight at 4°C. Complexes were isolated using protein A or protein G sepharose. The fractions were subjected to SDS-PAGE and transferred to PVDF membranes (Thermo Scientific, Waltham, MA, USA). The antibodies used for Western blot analysis were as follows: anti-EphA4 (EphA4, sc-135897), anti-β-tubulin (sc-5274, Santa Cruz Biotechnology, Santa Cruz, CA, USA), anti-phospho-c-Abl (C5240, Tyr-412, Sigma-Aldrich, St. Louis, MO, USA) mouse anti-phosphotyrosine (05-321, Millipore, Billerica, MA, USA), and anti-EphA4 phosphoTyr602 (EP2731, ECM Biosciences, Versailles, KY, USA) and WO-2 (MABN10, Millipore, Billerica, MA, USA)

### 2.6. Preparation of synaptoneurosomes

The synaptoneurosomes were obtained from adult rat hippocampi using a discontinuous Percoll gradient, employing modifications of the protocols described by Araya *et al*. (2007) and Villasana *et al*. (2006) [37], [38]. Briefly, the adult rat hippocampus was homogenised in ice-cold isolation medium I (320 mM sucrose; 10 mM HEPES, pH 7.4) with a Polytron (Ultra turrax T25 basic). The homogenate was centrifuged at 1,000 g for 10 min at 4°C; the pellet was discarded and the supernatant was centrifuged again under the same conditions. The final supernatant was centrifuged at 17,000 g for 20 min to obtain the crude synaptosomal pellet (P2 pellet). The P2 pellet was added to a Percoll (P1644, Sigma-Aldrich, St. Louis, MO, USA) density gradient (23%, 10% and 3% Percoll gradient). The tubes were centrifuged at 15,000 g for 20 min at 4°C. Synaptoneurosomes (band at the 10%: 23% Percoll interface) were collected and diluted (1∶1 vol/vol) in a 320 mM sucrose solution and centrifuged at 22,000 g for 20 min at 4°C.

### 2.7. Slice Preparation and Electrophysiology

Hippocampal slices were prepared according to standard procedures [39]. Briefly, transverse slices (350 μm) from the dorsal hippocampus from 2-month-old C57BL/6J mice were cut under cold artificial cerebrospinal fluid (ACSF: 124 mM NaCl, 2.6 mM NaHCO3, 10 mM D-glucose, 2.69 mM KCl, 1.25 mM KH2PO4 2.5 mM CaCl2, 1.3 mM MgSO4, and 2.60 mM NaH2PO4) using a Vibratome (Leica VT 1000 s, Germany) and incubated in ACSF for 1 h at room temperature. In all experiments, 10 μM picrotoxin (PTX) was added to suppress inhibitory GABA_A_ transmission. The slices were transferred to an experimental chamber (2 ml), superfused (3 ml/min, at 20–22°C) with gassed ACSF and visualised by trans-illumination with a binocular microscope (MSZ-10, Nikon, Melville, NY). To evoke field excitatory postsynaptic potentials (fEPSPs), we stimulated with bipolar concentric electrodes (Platinum/Iridium, 125 μm OD diameter, FHC Inc., Bowdoin, ME) and a stimulator (Axon 700 b, Molecular Devices, Sunnyvale, CA), connected to an isolation unit (Isoflex, AMPI, Jerusalem, Israel). The stimulation was in the *stratum radiatum* within 100–200 μm of the recording site. The paired pulse facilitation index was calculated by [(R2-R1)/R1], where R1 and R2 were the peak amplitudes of the first and second fEPSP, respectively. To generate LTP, we used theta burst stimulation (TBS) consisting of 5 trains of stimulus with an inter-train interval of 20 s. Each train consisted of 10 bursts at 5 Hz, and each burst had 4 pulses at 100 Hz. The recordings were filtered at 2.0–3.0 kHz, sampled at 4.0 kHz using an A/D converter, and stored with pClamp 10 (Molecular Devices). The evoked postsynaptic responses were analysed off-line, using analysis software (pClampfit, Molecular Devices) that allowed the visual detection of events, computing only those events that exceeded an arbitrary threshold.

### 2.8. Tissue Immunofluorescence

APPswe/PSEN1*Δ*E9 transgenic mice were obtained from Jackson Laboratory, sacrificed through CO_2_ inhalation and perfused transcardially with 4% paraformaldehyde in PBS. The brains were removed and post-fixed overnight at 4°C, followed by 20% and 30% sucrose in PBS at 4°C overnight. The brains were cut in 30-μm sagittal sections with a cryostat (Leitz 1900) at −20°C. The serial sections from all animal groups (n = 3/group) were processed in parallel for immunostaining using the same solutions to minimise variability in staining conditions. The sections were blocked with 3% BSA in PBS and incubated overnight at room temperature with rabbit anti-EphA4 phosphoTyr602 (EP2731, ECM Biosciences, Versailles, KY, USA). The primary antibody was detected by incubating for 1 hour with the corresponding Alexa-linked secondary antibodies Alexa-594 anti-mouse-IgG, Life Technologies, Carlsbad, CA, USA). For thioflavine-S (ThS) staining (Sigma Aldrich, St. Louis, MO, USA), the sections were incubated in ThS solution (0.025% in 50% ethanol) for 5 min prior to the immunofluorescence. Then, the sections were covered with Fluoromount and examined under a confocal microscope (Zeiss LSM 5 Pascal confocal microscope (NA 1.4)).

### 2.9 AβOs-FITC binding assay

HEK293 cells transfected with a pEphA4-Flag cDNA (pEphA4-Flag-pcDNA3 vector) or with pcDNA3-Flag (empty vector) as a control were plated on a 96-well-plate (Maxisorp, Nunc, IL, USA). Non-specific binding was blocked with 5% BSA-PBS. AβOs-FITC (Bachem, Torrance, CA, USA) were generated as described above and added to the immobilized cells incubating during 12 hours at 4°C. As controls, AβOs-FITC were pre-incubated either with KYL (90 μM) and ephrine-A3 ligand (9.5 μg/ml) for 4 hours at room temperature. Non-bound AβOs-FITC were removed using 0.05% Tween-PBS, and bound AβOs-FITC were detected using a Synergy H1 Hybrid Fluorometer (Biotek).

### 3.0 Statistical analyses

The results are presented as histograms showing the mean ± standard error. One-way ANOVA analyses of statistical significance in experiments with multiple conditions were performed using the GraphPad Prism software. Two-way ANOVA analyses were performed for grouped analyses using the GraphPad Prism software and Bonferroni post-hoc analyses. Student's t-tests were performed using SigmaPlot 10.0 software; for experiments with two conditions we used unpaired analysis.

## Results

### 3.1. AβOs induce c-Abl activation in hippocampal neurons

**Figure 1:**
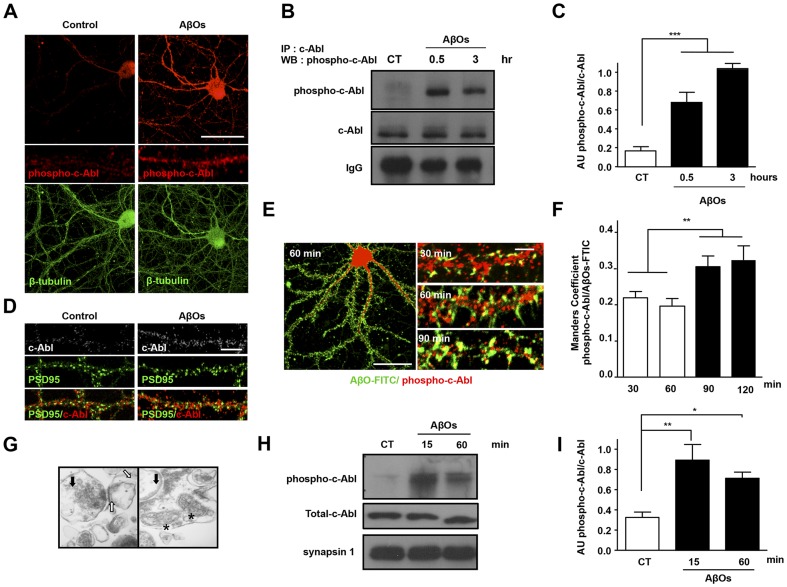
AβOs induce the c-Abl activation in hippocampal neurons. (A) c-Abl phosphorylated on tyrosine 412 (phospho-c-Abl; red) and β-tubulin (green). Hippocampal neurons (15 DIV) treated with 3 μM AβOs for 3 hours showed an increase in phospho-c-Abl immunoreactivity in the cell body and dendritic processes. Scale bar, 10 μm. (B) c-Abl was immunoprecipitated (IP) and then analyzed by western blotting (WB) for phospho-c-Abl and total c-Abl. IgG indicates the heavy chain of the antibodies used for immunoprecipitation. (C) The histogram shows densitometric analysis of phosphorylated c-Abl normalized to total immunoprecipitated c-Abl (average values ± standard error; ***p<0.001 by one-way ANOVA; n = 3). (D) Immunofluorescence labeling for c-Abl (gray) of 21 DIV hippocampal neurons treated with 3 μM AβOs 6 hours and postsynaptic protein PSD95 (green). Scale bar, 5 μm. (E) Cultured hippocampal neurons (21 DIV) were treated with 5 μM AβOs-FITC for 60 minutes and labeled for c-Abl phosphorylated on tyrosine 412 (phospho-c-Abl; red). Scale bar, 10 μm. Images of dendrites treated with AβOs-FITC for 30, 60 and 90 minutes labeled for phospho-c-Abl. (F) Histogram showing Manders overlap coefficient for AβOs-FITC and phospho-c-Abl at different times of treatment (**p<0.01 by one-way ANOVA; n = 15–20 neurites of 3 independent experiments). (G) Electron microscopy images of synatoneurosomes. The black arrows indicates vesicles accumulated in the active zone of presynaptic regions, asterisks indicate mitochondria, and white arrows indicate electron-dense regions corresponding to postsynaptic densities. (H) Immunoblot (phospho-c-Abl, total c-Abl and synapsin 1 (Syn)) of synaptoneurosomes exposed *in vitro* to 3 μM AβOs for 0, 15 and 60 minutes. (I) Densitometric analysis of phospho-c-Abl levels normalized to total c-Abl (AβOs for 15 minutes: **p<0.01 and for 60 minutes *p<0.05, by one-way ANOVA; n = 3).

We previously demonstrated that Aβ fibrils promote c-Abl activation. However, in those studies we did not investigate whether synaptic c-Abl is activated by AβOs, the main Aβ species that causes synaptic damage and loss. Therefore, we examined c-Abl activation in hippocampal neurons cultured for 21 days (21 DIV) that were exposed to AβOs prepared according to Sokolov *et al*. (2006) and Arimon *et al*. (2005) [36], [35]. AβOs were characterised through electron microscopy and western blot assays using WO-2, an antibody specific for β-amyloid peptide (Fig. S1A, B and C). c-Abl kinase activation is associated with its phosphorylation on tyrosine 412 [40]. Treatment of hippocampal neurons with AβOs for 1 hour significantly increased c-Abl phosphorylation on tyrosine 412 (phospho-c-Abl) in the neuronal cell body, nucleus and dendrites (Fig. 1A). Moreover, c-Abl immunoprecipitation followed by immunoblotting for phospho-c-Abl confirmed c-Abl activation as early as 30 minutes after AβOs treatment, and the activation persisted for at least 3 hours (Fig. 1B and C). These results show that AβOs induced c-Abl kinase activation in hippocampal neurons. A more detailed analysis showed that the increase phospho-c-Abl in dendrites was distributed in discrete structures, indicating synaptic localisation (Fig. 1A amplification). Previously, we have described that c-Abl is enriched in postsynaptic regions and that interacts with PSD95 [8]; here, we show that the c-Abl signal partially colocalises with PSD95, suggesting synaptic c-Abl activation by AβOs (Fig. 1D).

In vertebrates, the Abl family has two members, c-Abl and Arg (Abl-related gene) kinases, which are highly conserved with overlapping and distinct functions [7]. Interestingly, we did not detect an increase in tyrosine phosphorylation of Arg immunoprecipitated from hippocampal neurons treated with AβOs (Fig. S2A) [41].

AβOs bind to PSD95- and NMDA receptor-positive excitatory synapses but not to GABA receptor-positive inhibitory synapses [22]. To examine the spatial relationship between AβOs bound to synaptic regions and c-Abl activation in more detail, AβOs labelled with fluorescein isothiocyanate (AβOs-FITC) were used to examine AβOs localisation with respect to phospho-c-Abl. This analysis revealed that activated c-Abl kinase colocalises with or is in close proximity to the bound AβOs-FITC (Fig. 1E). In addition, we found that the colocalisation of AβOs-FITC with phospho-c-Abl increases in a time-dependent manner (Fig. 1E and F). Analysis using the Manders colocalisation coefficient (Fig. 1F) showed a significant difference between 30-60 minutes and 90–120 minutes of AβOs exposure. These results support that AβOs binding promotes c-Abl activation in synaptic structures.

As an alternative approach to evaluate synaptic c-Abl activation driven by AβOs, we used preparations of metabolically active synaptoneurosomes obtained from adult rat hippocampus [37], [38]. These preparations are enriched in sealed presynaptic structures anchored to sealed postsynaptic regions (Fig. 1G). c-Abl phosphorylation at tyrosine 412 was significantly increased in synaptoneurosomes treated with AβOs for 15 and 60 minutes (Fig. 1H and I). Altogether, these results suggest that AβOs can locally activate synaptic c-Abl, which may participate in the signalling events that mediate the synaptic damage caused by AβOs.

### 3.2. AβOs induce EphA4 receptor activation in hippocampal neurons

The c-Abl kinase is an important transducer of extracellular signals [42]. Among the neuronal receptors that could modulate c-Abl activity, EphA4 is an interesting candidate because it is enriched in the hippocampus, and its activation participates in synapse elimination through Cdk5 activation [27]. Interestingly, we previously found that the cytoskeletal changes associated with c-Abl activation by Aβ fibrils involve Cdk5 [11]. EphA4 is highly expressed in dendritic spines of hippocampal neurons, and can be activated under physiological conditions by binding to the ephrin-A3 ligand expressed in glial cells [28], [43].

**Figure 2:**
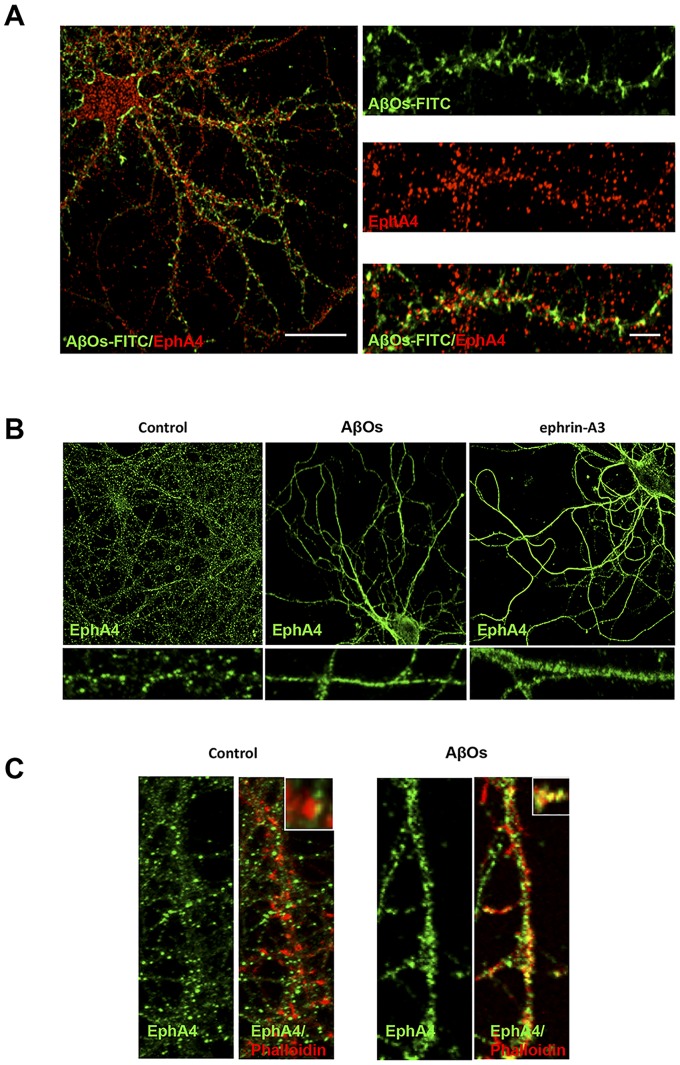
AβOs colocalize with the EphA4 receptor and induce changes in its subcellular localization. (A) Cultured hippocampal neurons (21 DIV) were treated with 5 μM AβOs-FITC (green) for 0.5–1 hours and immunolabeled for EphA4 (red). Scale bars, 10 μm in the left panel and 5 μm in the right panels. (B) Hippocampal neurons (21 DIV) were treated with 3 μM AβOs or 9.5 μg/ml ephrin A3 Fc as a soluble EphA4 ligand for 1 hour or left untreated as a control. The neurons were then immunolabeled with an EphA4 antibody (green; B) or double-labeled with an EphA4 antibody and phalloidin-TRITC (red; C).

Next, we studied whether AβOs induce EphA4 receptor activation in hippocampal neurons. A short time after the addition of AβOs to cultures (0.5 or 1 hours), AβOs were found in close proximity to EphA4-positive post-synaptic regions (Fig. 2A). Longer AβO treatments caused EphA4 relocalisation from dendritic spines to the dendritic shaft, similar to the changes induced by treatment with the ephrin-A3 Fc ligand (Fig. 2B and C).

**Figure 3:**
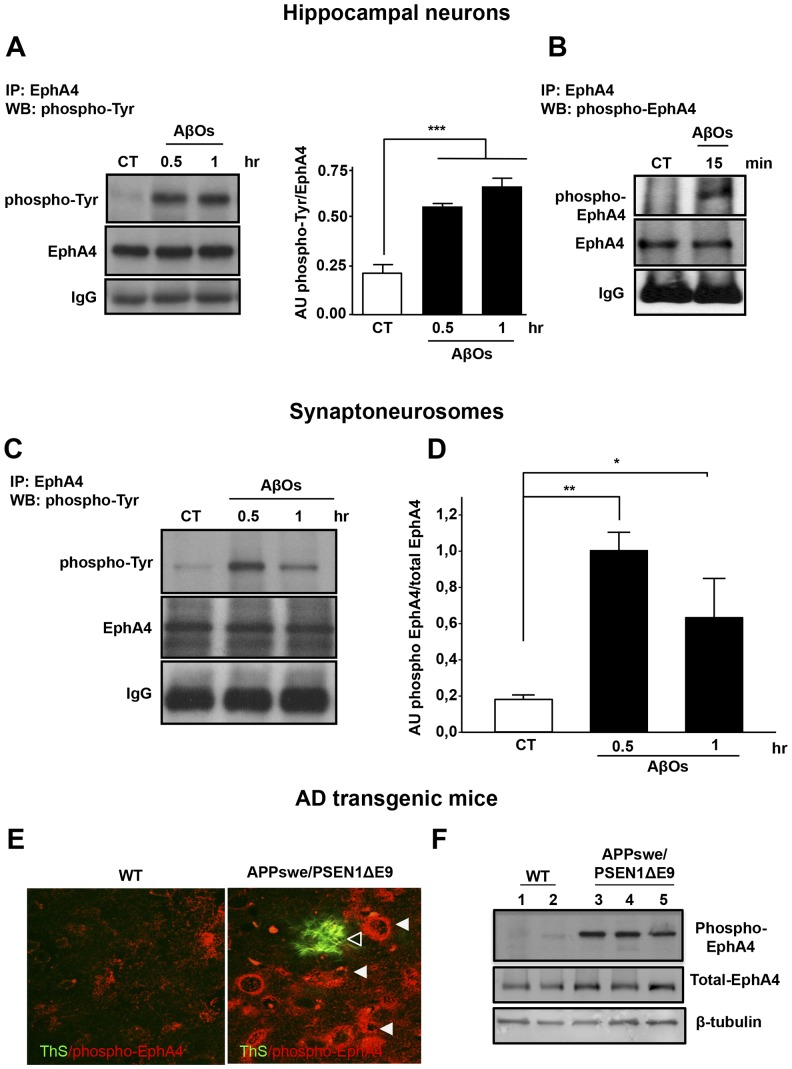
EphA4 is activated in hippocampal neurons treated with AβOs and in Alzheimer's disease transgenic mice. (A) Cultured hippocampal neurons (15 DIV) were treated with 3 μM AβOs for 0.5 and 1 hour EphA4 was immunoprecipitated and then analyzed by immunoblotting with an anti-phosphotyrosine antibody or (B) anti-phospho-EphA4 Tyr 602, shows that the quick response of EphA4 (15 minutes). The histogram shows densitometric analysis of phosphotyrosine levels normalized to total EphA4 levels (mean ± standard error; ***p<0.001 by one-way ANOVA; n = 3). (C) Synaptoneurosomes were treated with 3 μM AβOs for 0.5 and 1 hour. EphA4 was immunoprecipitated and then analyzed by immunoblotting using an anti-phosphotyrosine antibody. (D) The histogram shows densitometric analysis of phosphotyrosine levels at 0,5 and 1 hour normalized to total EphA4 levels (AβOs 0.5 hour **p<0.01, AβOs 1 hour *p<0.05 by Student's *t*-tests; n = 3). (E) Immunofluorescence showing phospho-EphA4 (p-EphA4) (red) and thioflavine-S (ThS) staining (green) in APPswe/PSEN1*Δ*E9 transgenic and wild-type (WT) hippocampi. Empty arrowheads show phospho-EphA4-positive neurons and white arrowheads show amyloid deposits stained with thioflavine-S (ThS). (F) Western blot of either wild-type or APPswe/PSEN1*Δ*E9 transgenic mouse hippocampus homogenates blotted against phospho-EphA4 Tyr 602 and total EphA4. Wild-type mouse lines 1–2 and APPswe/PSEN1*Δ*E9 transgenic mice lines 3–5.

Moreover, treatment of hippocampal neurons with AβOs induced a significant increase in EphA4 activation. We observed rapid phosphorylation of the receptor on tyrosine 602 after 15 minutes of AβO treatment (Fig. 3A, B), while total levels of EphA4 remained constant (Fig. S3A). More importantly, AβOs activated EphA4 in synaptoneurosomes (Fig. 3C and D), indicating that AβOs can locally activate synaptic EphA4.

Furthermore, we analysed brain slices from APPswe/PSEN1*Δ*E9 transgenic mice, an AD mouse model, to evaluate the phosphorylation of EphA4 at Tyr-602 *in vivo*. Immunofluorescence analysis of APPswe/PSEN1*Δ*E9 hippocampal slices showed an increased number of phospho-EphA4-positive cells, especially around the thioflavin-S-positive amyloid plaques compared to wild-type mice (Fig. 3E). Accordingly, we observed EphA4 activation in AD transgenic mice. Western blot analysis showed increased levels of EphA4 phosphorylation on 602 tyrosine in APPswe/PSEN1*Δ*E9 brain extracts compared to wild type brain homogenates, but no differences were observed in the total levels of EphA4 (Fig. 3F; n = 3).

### 3.3. EphA4 is required for AβO-induced c-Abl phosphorylation

**Figure 4:**
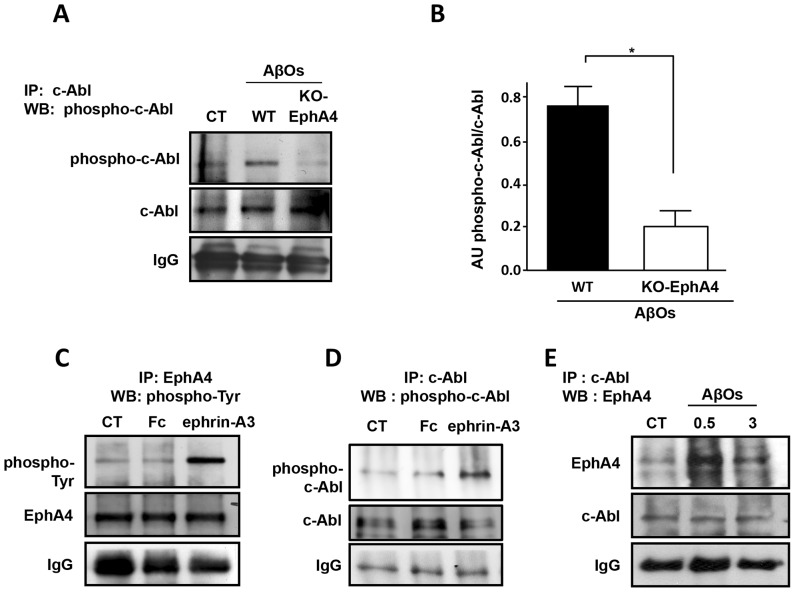
EphA4 induces c-Abl activation in response to AβOs. (A) Primary cultures of wild-type (WT) and EphA4 knockout (KO) hippocampal neurons were treated with AβOs for 60 minutes. c-Abl immunoprecipitates were probed by immunoblotting with anti-phospho c-Abl Tyr-412 antibody. (B) The histogram shows densitometric analysis of phospho-c-Abl levels normalized to total c-Abl in EphA4 WT and KO neurons treated with AβOs (mean ± standard error, *p<0.05 by one-way ANOVA; n = 3). C) Hippocampal slices from adult mice were treated for 90 minutes with 9.5 μg/ml ephrin-A3 ligand Fc or Fc as a control. EphA4 was immunoprecipitated, and the immunoprecipitates were probed by immunoblotting with an anti-phosphotyrosine antibody and reprobed for EphA4. (D) The hippocampal slices were stimulated as in C. c-Abl was immunoprecipitated, and immunoblotting was performed to detect anti-phospho c-Abl Tyr-412 antibody and reprobed for c-Abl. (E) Cultured hippocampal neurons (15 DIV) were treated with 3 μM AβOs for 0.5 or 3 hours, and c-Abl was then immunoprecipitated (IP). Immunoblotting was performed to detect EphA4 and c-Abl and showed that AβOs promote EphA4 association with c-Abl.

Using c-Abl immunoprecipitation followed by c-Abl phosphorylation on tyrosine 412 immunoblotting, we observed that treatment with AβOs for 1 hour increases c-Abl phosphorylation in wild-type EphA4 hippocampal neurons but not in EphA4-knockout hippocampal neurons (Fig. 4A and B), demonstrating that EphA4 is required for c-Abl activation by AβOs. As a control experiment, we confirmed that the stimulation of hippocampal neurons with the ephrin-A3-Fc ligand induces EphA4 tyrosine phosphorylation (Fig. 4C) accompanied by increased c-Abl phosphorylation on tyrosine 412 (Fig. 4D), as previously demonstrated by Yu *et al* (2001) [19]. Thus, c-Abl is activated downstream of EphA4 in hippocampal neurons exposed to AβOs. In addition, we detected an increased interaction between EphA4 and c-Abl in neurons treated with AβOs (Fig. 4E), indicating that AβOs promote c-Abl recruitment to EphA4, activating EphA4/c-Abl signalling. These results show that in hippocampal neurons, AβO binding to synaptic regions of dendrites triggers EphA4/c-Abl signalling (See, Fig. S2B), and suggest that EphA4 could contribute to AβO binding to synapses and to the downstream signalling associated with synapse dysfunction and loss.

Consistent with this observation and using different AβO-FITC concentrations (0–50 μM) in binding assay (Fig. S3B, C and D), we observed that HEK293 cells overexpressing EphA4-Flag display a one-site binding behaviour with higher binding of AβO-FITC compared to control cells.

### 3.4. EphA4/c-Abl signalling inhibition prevents synaptic damage induced by AβOs

**Figure 5:**
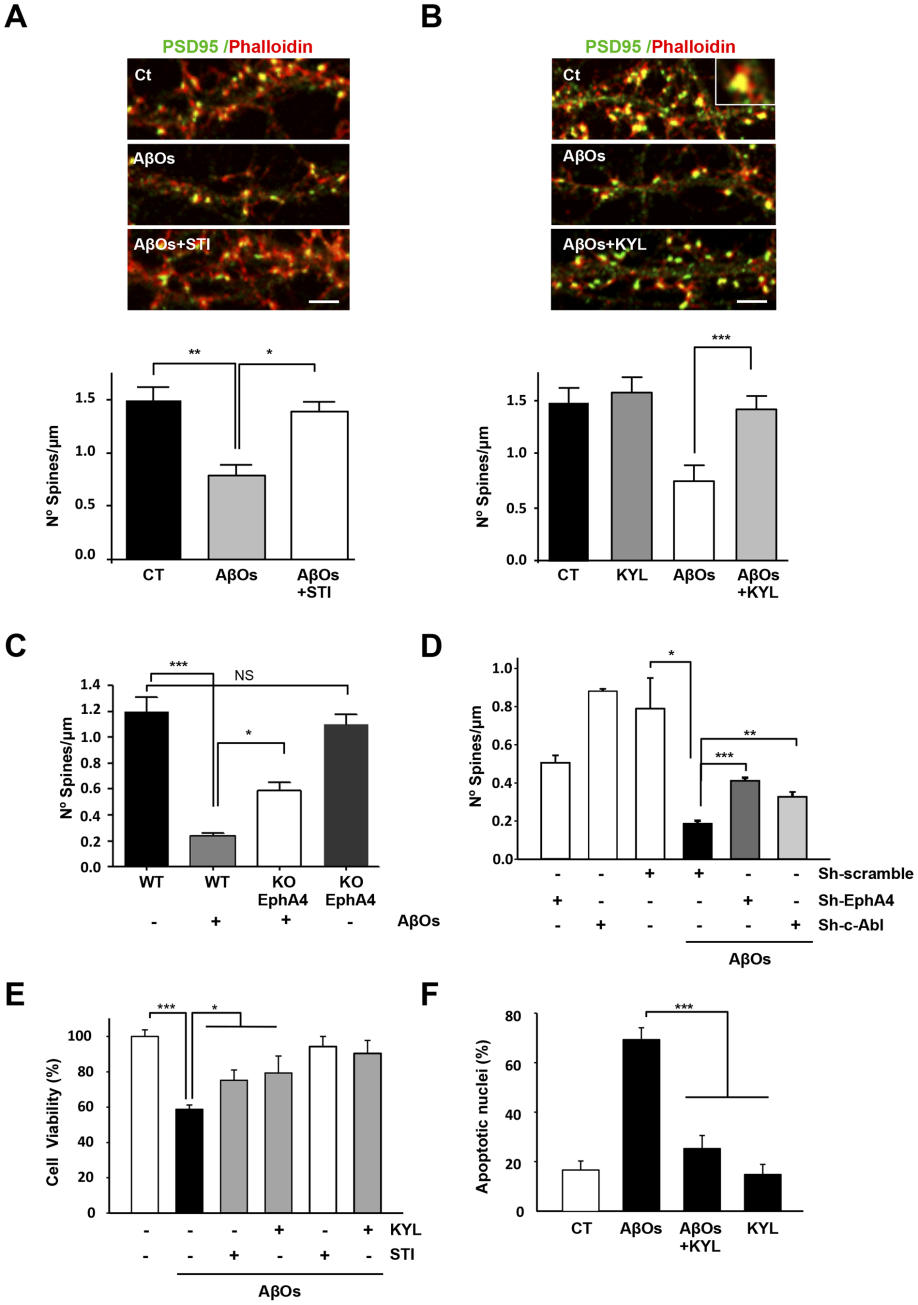
Inhibition of the EphA4-c-Abl pathway decreases the loss of dendritic spines and apoptosis induced by AβOs. (A) Cultured hippocampal neurons (21 DIV) exposed for 5 hours to 3 μM AβOs with and without STI (5 μM) and (B) KYL (60 μM). The confocal images show F-actin labeled with phalloidin (red) and immunofluorescence labeling for PSD95 (green). Scale bar, 5 μm. Densitometric analysis (A. **p<0.01 for control versus AβOs; *p<0.05 for AβOs versus AβOs + STI. B. ***p<0.001 AβOs versus AβOs + KYL. By one-way ANOVA; n = 15–20 neurites of 3 independent experiments). All graphics show the average values ± standard error. (C) Quantification of dendritic spines in cultures of wild-type (EphA4^+/+^, WT) or EphA4 knockout (EphA4^-/-^, KO) hippocampal neurons (15 DIV) exposed to AβOs for 5 hours (*p<0.05, ***p<0.001, no significant (NS) by one-way ANOVA; n = 15–20 neurites of 3 independent experiments). (D) Quantification of dendritic spines in neurons transfected with pGFP, sh-EphA4, sh-c-Abl, scramble RNA EphA4 and c-Abl (SC) and treated with AβOs for 5 hours (*p<0.05 for SC versus pGFP+ AβOs; ***p<0.001 for pGFP+ AβOs verus Sh-EphA4+ AβOs or sh-c-Abl+ AβOs **p<0,01). (E) Cell viability assay in primary cultures of hippocampal neurons treated with AβOs and/or 15 or 30 μM KYL peptide for 24 hours (AβOs versus AβOs+ STI and AβOs+ KYL *p<0,05; n = 3). (F) Quantification of apoptotic nuclei in primary cultures of hippocampal neurons treated with AβOs and/or 30 μM KYL for 24 hours.

Next, we evaluated whether c-Abl and EphA4 signalling activation caused by AβOs may contribute to AβO-induced dendritic spine loss. First, to determine whether c-Abl activity is involved in AβO-induced dendritic spine loss, we co-treated hippocampal neurons at 21 DIV with AβOs and the c-Abl inhibitor STI. The number of dendritic spines was quantified after 5 hours using phalloidin-TRITC and PSD95 immunodetection (Fig. 5A). AβO treatment induced a significant decrease in spine density (0.79±0.089 spines/μm). Interestingly, when neurons were treated with AβOs together with STI, the number of dendritic spines was not significantly affected (1.41±0.071 spines/μm; Fig. 5A) and was significantly higher than in neurons treated with AβOs. In addition, we used a 12 amino acid-long peptide called KYL, which specifically targets the ligand-binding pocket of the EphA4 receptor and inhibits ephrin ligand binding, thus preventing EphA4 activation [33], [34], [44]. We found that 30 μM KYL significantly inhibited dendritic spine loss induced by AβO treatment (Fig. 5B). Therefore, EphA4/c-Abl pathway inhibitors prevent spine loss induced by AβOs, strongly suggesting that EphA4/c-Abl signalling contributes to AβO synaptotoxicity.

Next, we examined whether EphA4 expression is required for the AβO-induced decrease of dendritic spine density. Exposure of hippocampal neurons from wild-type mice to AβOs significantly decreased spine density (from 1.33±0.08 spines/μm in control neurons versus 0.24±0.01 spines/μm in AβO-treated neurons; Fig. 5C). Spine density in EphA4-knockout neurons under control conditions was slightly lower than wild-type neurons; however, this difference was not statistically significant (NS). Interestingly, the spine density decrease induced by AβOs was significantly less pronounced in the EphA4-knockout mouse (0.54±0.069 spines/μm; Fig. 5C, Fig. S4A). It has been described that EphA4 knockout neurons have morphological irregularities in dendritic spines [28]; indicating that EphA4 knockout neurons have alterations in dendritic spine development [28]. In order to discard that those developmental alterations are concealing the real effect, we transfected hippocampal neurons with a short hairpin for the EphA4 receptor (sh-EphA4 plus pGFP) at 15 DIV to induce its knockdown in neurons that with mature dendritic spines. At 21 DIV the neurons were treated with AβOs and we evaluated AβO-induced dendritic spine loss after 5 hours. Although the number of dendritic spines in transfected neurons was lower than in non-transfected neurons (Fig. 5A and D, Fig. S4B), the decrease in dendritic spines induced by AβOs was significantly inhibited in sh-EphA4 and sh-c-Abl neurons (Fig. 5D, Fig. S4B; sh-scramble+AβOs, 0.186 spines/μm vs. sh-EphA4+AβOs, 0.410 and sh-c-Abl+AβOs 0.312 spines/μm). This key result supports the idea that the EphA4 receptor and c-Abl are required for the signalling that results in the loss of synapses.

In AD, synaptic loss precedes neuronal death and longer AβOs exposures induce neuronal apoptosis, indicating that these damages could share signalling mechanisms. Therefore, we examined whether blocking EphA4 and c-Abl activity would prevent AβO-induced neuronal death. AβO treatment for 24 hours decreased neuronal viability by 50%, while a concomitant inhibition of EphA4 with 30 μM KYL peptide or c-Abl inhibition with STI (5 μM) significantly prevented neuronal death (Fig. 5E). KYL also decreased the number of apoptotic nuclei in neurons treated with AβOs (Fig. 5E and F). Thus, EphA4 signalling activated by AβOs may participate in early synaptic loss and later, in neuronal death.

### 3.5. STI and KYL block the effect of AβOs allowing LTP induction of hippocampal slices from wild-type mice

**Figure 6:**
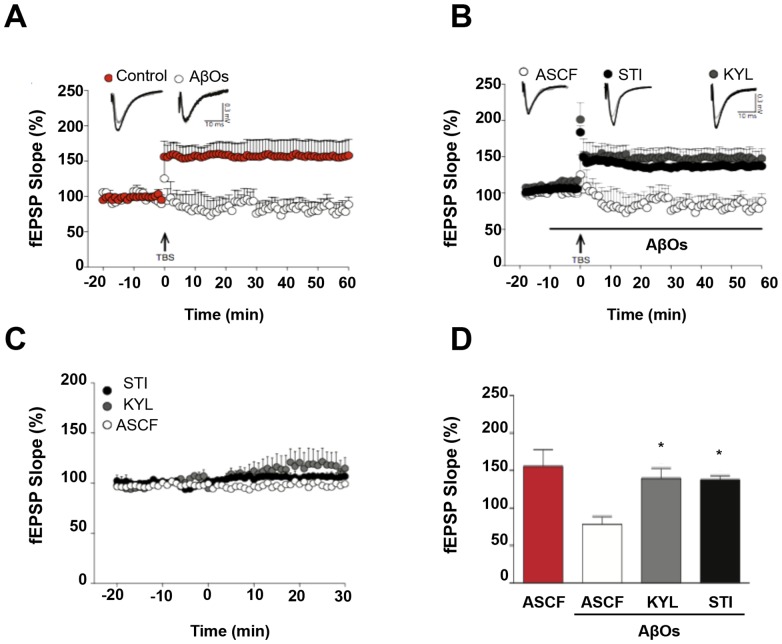
STI and KYL block the AβO effect in vitro, allowing for the induction of LTP of the hippocampal slices from wild-type mice. (A) Hippocampal slices were exposed to ACSF (red) or AβOs (1 μM, white); arrow indicates the time of TBS and the plot show the fEPSP slope at different times. (B) Hippocampal slices were exposed to AβOs and STI (5 μM, black) or KYL (30 μM, gray), arrow indicates the time of TBS and the plot show the fEPSP slope at different times. (C) Inhibitors of c-Abl and EphA4 does not alter amplitude of basal fEPSP. Hippocampal slices were exposed to ACSF (white), STI (5 μM, black) or KYL (30 μM, gray) in basal conditions without stimulation. (D) Histogram showing Plot of fEPSP electrical recordings.

It has been reported that AβO treatment blocks induction of long-term potentiation (LTP) in hippocampal CA3-CA1 transmission [39], [45]. We performed recordings in hippocampal slices and observed that AβOs prevent LTP induction (Fig. 6A). However, this effect was antagonized completely when brain slices were pre-incubated either with STI (5 μM) or KYL (30 μM) before the addition of AβO (Fig. 6B and D), indicating that the inhibition of EphA4/c-Abl signalling protects synaptic plasticity in the hippocampal CA3-CA1 transmission against AβOs. As a control, STI and KYL alone did not change basal transmission in CA1 (Fig. 6C).

## Discussion

Our results show that the EphA4/c-Abl signalling pathway participates in AβO-induced synaptic effects. The major findings of this work are: i) in neurons, AβO exposure induces activation of c-Abl and EphA4 at the synapse; ii) EphA4 activation by AβOs is linked to c-Abl activation; and iii) EphA4 as well as c-Abl are required for LTP blockage and dendritic spine number reduction induced by AβOs.

### i) AβO exposure induces the synaptic activation of c-Abl and EphA4

Here, we show that AβOs induce c-Abl activation. AβOs have been described as the most neurotoxic species, capable of inhibiting long-term potentiation (LTP), enhancing long-term depression (LTD) and reducing dendritic spine density [62], [63], [64]; while Aβ fibrils have been associated with neuronal apoptosis and cytoskeletal alterations. Still, Aβ fibrils and AβOs might share signalling mechanisms for inducing neuronal damage. In fact, AβOs promote major cytoskeleton alterations and neuronal death at higher concentrations or longer exposure times. Indeed, herein we found that longer treatments with AβOs cause neuronal apoptosis. Taking into account the main role of AβOs on synaptic changes and our previous report that c-Abl is localised in synaptic regions [8], we hypothesise that the observed AβO induced c-Abl activation (Fig. 1D) could be linked to a local synapse signalling involved in earlier synaptic alterations as well as later, in neuronal death and cytoskeleton alterations described previously by our group [9], [10], [11].

We show that AβOs induce EphA4/c-Abl signalling activation locally, in the synapses. We detected c-Abl activation in synaptic regions positive for AβOs-FITC and in synaptoneurosomes treated with AβOs, indicating that c-Abl activation occurs in synapses where AβOs are bound [48]. In addition to c-Abl activation, we found that AβOs activate the synaptic tyrosine kinase receptor EphA4 (Fig. 3A). EphA4 has key roles in patterning and morphogenesis [65], it has been implicated in axon pathfinding and mediates growth cone collapse [59] and dendritic spine retraction [27]. EphA4 is present in synapses, and in recent years, it has been shown to be involved in synaptic remodelling and plasticity [56], [55]. We detected increased EphA4 tyrosine phosphorylation in cultured neurons exposed to AβOs, in isolated synaptoneurosomes and, in vivo, in the brains of Alzheimer's transgenic mice. Moreover, we observed that AβO binding to synaptic sites caused EphA4 activation and redistribution in the dendrites, similar to the changes induced by its ligand ephrin-A3 (Fig. 2B). This is the first time that a role for EphA4 has been described in relation to AβO synaptic effects. This is consistent with the report that patients with early signs of AD have increased EphA4 receptor mRNA levels, and that single nucleotide polymorphisms in the EPHA4 gene are associated with AD susceptibility [66].

In addition, the EphA4 receptor has been described as involved in amyotrophic lateral sclerosis [68] and as a modulator of; ischemia-reperfusion [44], [70], contusive spinal cord injury [67], [69] and experimental autoimmune encephalomyelitis progression [70], supporting a role for EphA4 signalling in neuronal damage and neurodegeneration.

Several previous reports link AβO toxicity to other signalling pathways including NMDA and AMPA glutamate receptors, the EphB2 receptor, the α7 nicotinic acetylcholine receptor (α7nAChR), the insulin receptor and the cellular prion protein (PrPc) [22], [23], [24], [46], [47], [48], [49], [50], [51]. The contribution of these receptors to AβO pathological effects is controversial, suggesting that multiple receptors may be involved in AβO-induced synaptic damage. According to the literature, the synaptotoxicity induced by AβOs is not a consequence of a single receptor. Therefore, it is necessary to hierarchize the contributions of each receptor to synaptic damage and the downstream signalling pathways involved. Perhaps, many of the signalling pathways initiated by these receptors converge into common downstream targets that are ultimately responsible for dendritic spine retraction and loss. In this context, the c-Abl kinase has been implicated in downstream signalling of several of the aforementioned receptors and interestingly c-Abl is directly involved in actin regulation. It has been shown that active c-Abl decreases NMDA-evoked currents [52], and also, c-Abl has been associated with the insulin receptor signalling [53]. Furthermore, it has been shown that EphA4 induces AMPA receptor downregulation in homeostatic plasticity [54] and regulates LTP through glial glutamate transport [43], [55]. The EphA4 signalling regulates synaptic plasticity and promotes spine maturation in cortical neurons [56], [57]. Recently, it was described that EphA4 activity also mediates homeostatic scaling down of synaptic strength via activation of Cdk5 [58]. During brain development EphA4 mediates growth cone collapse [59] and pruning of dendritic spines [28], [60], [27]. EphA4 activation by ephrin-A3 induces spine retraction, and inhibition of the interaction between ephrin/EphA4 alters spine shape and organisation in hippocampal slices [28] and in activity-dependent synaptic wiring in the mouse cerebellar cortex [61]. Further studies are required to determine the contribution of the Eph4/c-Abl signalling pathway to AβO-induced synaptic toxicity and its relation to other signalling pathways already described.

### ii) EphA4 activation by AβOs is link to c-Abl activation

Our results show that c-Abl tyrosine kinase is activated in response to AβOs and that EphA4 is upstream of c-Abl activation. Interestingly, c-Abl activation by AβOs was not detected in EphA4-knockout neurons, strongly supporting EphA4 as a key receptor responsible for AβOs induced-c-Abl activation. In addition, AβO treatment leads to increased EphA4-c-Abl interactions (Fig. 4E). It has been reported that the SH2 domains of c-Abl bind to tyrosine-phosphorylated motifs in the juxtamembrane region of EphA4 and that activated EphA4 causes tyrosine phosphorylation of c-Abl, and vice versa [19].

### iii) EphA4 and c-Abl signalling are required for AβOs induced dendritic spine loss and LTP blocking

We observed that EphA4/c-Abl signalling inhibition prevented AβO synaptotoxicity (dendritic spine loss and the blockage of LTP), using different approaches: i) pharmacological inhibition using STI (STI571, c-Abl inhibitor) and KYL (EphA4 inhibitor) inhibitors; ii) c-Abl and EphA4 protein expression reduction using shRNAs; and iii) using EphA4 receptor knockout neurons. STI and KYL inhibitors prevented AβO-induced dendritic spine loss. Although the absence of EphA4 did not completely rescue the dendritic spine loss, EphA4-knockout neurons show a significant decrease in AβO-induced dendritic spine loss. Furthermore, protection was observed in mature neurons (with established synapses) transfected with either c-Abl shRNA or EphA4 shRNA. According to these results, and using different approaches to modulate the signalling of EphA4/c-Abl in the presence of AβOs, we conclude that the EphA4/c-Abl signalling pathway mediates the synaptotoxicity and neuronal damage induced by AβOs.

Our results show that AβOs bind to cells overexpressing EphA4 suggesting that EphA4 could be a new AβOs receptor (Fig. S3B). The inhibition of AβO binding and synaptic protective effects mediated by KYL suggests that AβOs may bind to the EphA4 [34], leading to receptor activation. This mechanism would be different from the one proposed for EphB2, which involves the binding of AβOs to the fibronectin type III repeats of the receptor [24], promoting the EphB2 loss of function; receptor involved in synapses maturation and stabilization.

In conclusion, we have identified a new AβO-triggered signalling pathway that mediates in synaptic elimination and neuronal apoptosis. EphA4-c-Abl activation may be a key pathway that is altered in the early stages of Alzheimer's disease, when synaptic damage begins to occur. Our results sustain that AβOs binding to synaptic regions leads to EphA4 receptor activation and to the downstream activation of c-Abl. In turn, c-Abl may join EphA4 downstream effectors, triggering signalling events that drive actin cytoskeleton rearrangements, promoting spine loss and causing cell death in more advanced disease stages. EphA4/c-Abl signalling pathway could be a relevant in the early cognitive decline observed in Alzheimer's disease. Further studies are required to determine the contribution of this pathway and its relationship to other signalling pathways involved in early stages of the disease. The use of pharmacological inhibitors such as STI and KYL could be valuable therapeutic tools for preventing dendritic spine loss and inhibition of LTP induction triggered by AβOs in Alzheimer's disease.

## Supporting Information

Figure S1 A) Aβ1-42 oligomers were prepared by dissolving 1 mg of Aβ1-42 in 2 mL of 1,1,1,3,3,3 hexafluoro-2-propanol (HFIP) to freeze-dry. Then, the resulting lyophilized peptide was resuspended in H_2_O at a 200 μM concentration. Aβ1-42 aliquots when incubated at 4°C O/N, aggregates into small oligomers (mostly dimers and trimers) (Lane 1). When incubated at 37°C with shaking, Aβ1-42 aliquots aggregates mostly as trimers and dodecamers (Lane 2). Samples were run on Tris-Tricine gels on denaturant conditions and western blot was performed using 4G8 anti-Aβ antibody. (B) Characterization of AβOs 1-42. In electron microscopy shows globular species (white arrows) at time cero. In the box of AβOs we observed species of 20 nm described in the literature as AβOs (empty arrows). (C) Cell viability assay in primary cultures of hippocampal neurons treated with AβOs or Aβ-fibers at different concentration for 24 hours (n = 3).
(TIF)

Figure S2 (A) Cultured hippocampal neurons (15 DIV) were treated with 3 μM AβOs for 0.5 and 3 hours. Arg was immunoprecipitated and then analyzed by immunoblotting with an anti-phosphotyrosine antibody. (image representative of three independent experiments). (B) Hippocampal neurons were treated for 90 minutes with AβOs-FITC (green) and immunolabeled for EphA4 (red) and c-Abl (blue). The circles show examples of co-localization of the 3 labels. Scale bar, 5 μm.
(TIF)

Figure S3 (A) Cultured hippocampal neurons (7 DIV) were treated with 5 μM AβOs for 0.5 to 24 hours. Immunoblot showing total EphA4 levels (B) HEK293 cells overexpressing EphA4-Flag show increase binding of AβOs-FITC compared to controls cells (empty vector), while pre-incubation with the specific inhibitor KYL or the ephrin-A3 ligand of EphA4 receptor displaced AβOs-FITC binding. The data was fitted to a one site-specific binding curve (dash line) obtaining a Kd of 22 μM for AβOs-FITC binding in cells overexpressing EphA4 receptor. Data was analized by two-anova followed by Bonferroni's test. (**p<0.01, *** p<0.001) (C). HEK293 cells that overexpress EphA4-Flag or pcDNA-Flag. Immunoblotting was performed to detect EphA4 and Flag. (D) Immunofluorescence labeling for the Flag epitope (red) and AβOs-FITC labeling (green) of pcDNA-Flag and EphA4-Flag expressed in HEK293 cells. Representative confocal microscopy images are shown.
(TIF)

Figure S4 (A) Dendritic spines of wild-type (EphA4^+/+^, WT) or EphA4 knockout (EphA4^-/-^, KO) cultures of hippocampal neurons (15 DIV) exposed to AβOs for 5 hours. Confocal images showing phalloidin-TRITC staining (red). Scale bar, 5 μm. (B) Neurons transfected with pGFP, sh-EphA4, sh-c-Abl (green), scramble RNA EphA4 and c-Abl (SC) and treated with AβOs for 5 hours. Confocal images showing phalloidin-TRITC (red) and GFP (green) Scale bar, 5 μm.
(TIF)
