# Supplementary figures and images for: EphA4 Activation of c-Abl Mediates Synaptic Loss and LTP Blockade Caused by Amyloid-β Oligomers
Source: PLoS One. 2014 Mar 21;9(3):e92309. doi: 10.1371/journal.pone.0092309 (PMC3962387; doi:10.1371/journal.pone.0092309)

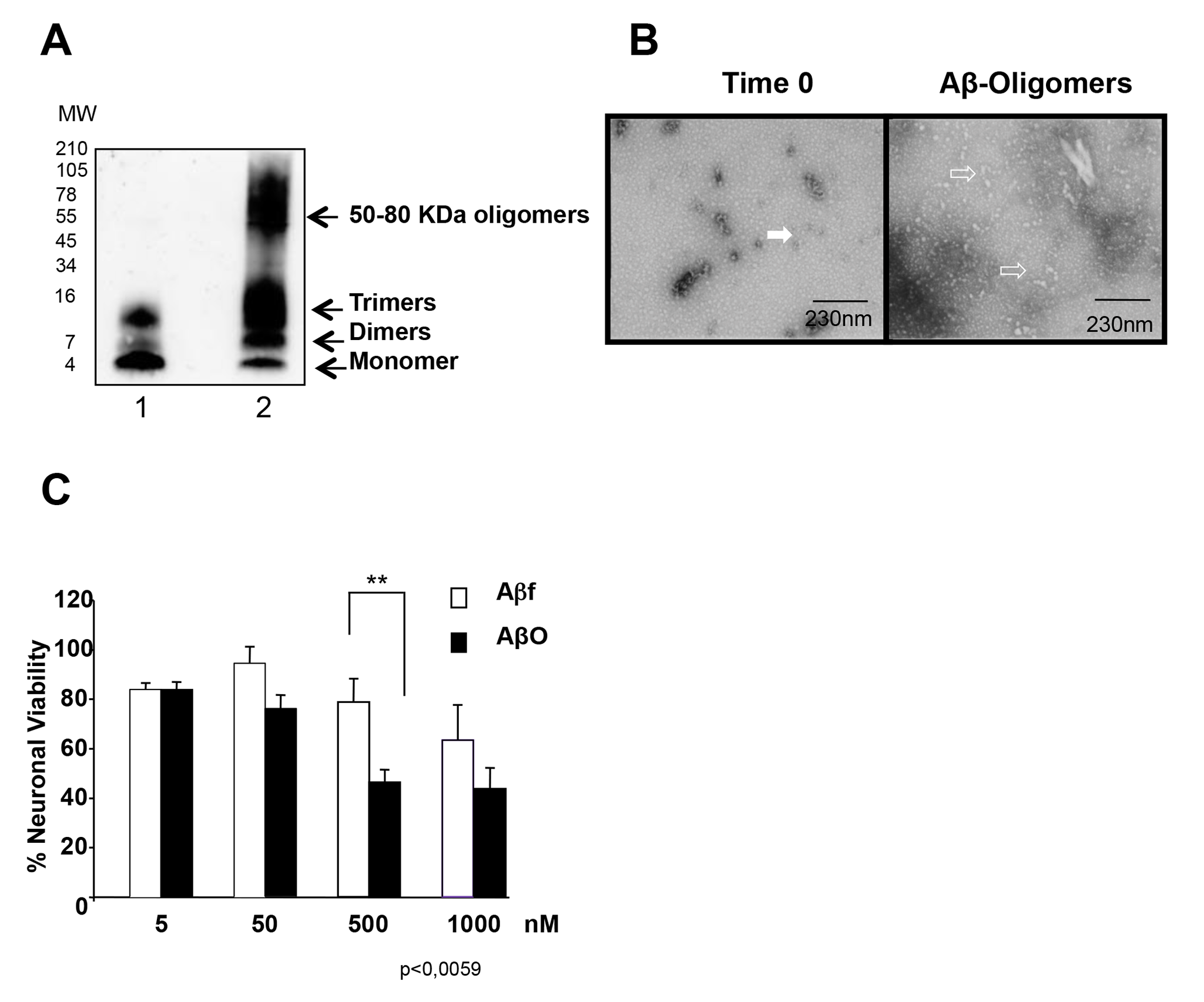

Supplement: Figure S1 — A) Aβ1-42 oligomers were prepared by dissolving 1 mg of Aβ1-42 in 2 mL of 1,1,1,3,3,3 hexafluoro-2-propanol (HFIP) to freeze-dry. Then, the resulting lyophilized peptide was resuspended in H2O at a 200 μM concentration. Aβ1-42 aliquots when incubated at 4°C O/N, aggregates into small oligomers (mostly dimers and trimers) (Lane 1). When incubated at 37°C with shaking, Aβ1-42 aliquots aggregates mostly as trimers and dodecamers (Lane 2). Samples were run on Tris-Tricine gels on denaturant conditions and western blot was performed using 4G8 anti-Aβ antibody. (B) Characterization of AβOs 1-42. In electron microscopy shows globular species (white arrows) at time cero. In the box of AβOs we observed species of 20 nm described in the literature as AβOs (empty arrows). (C) Cell viability assay in primary cultures of hippocampal neurons treated with AβOs or Aβ-fibers at different concentration for 24 hours (n = 3). (TIF) [file pone.0092309.s001.tif]

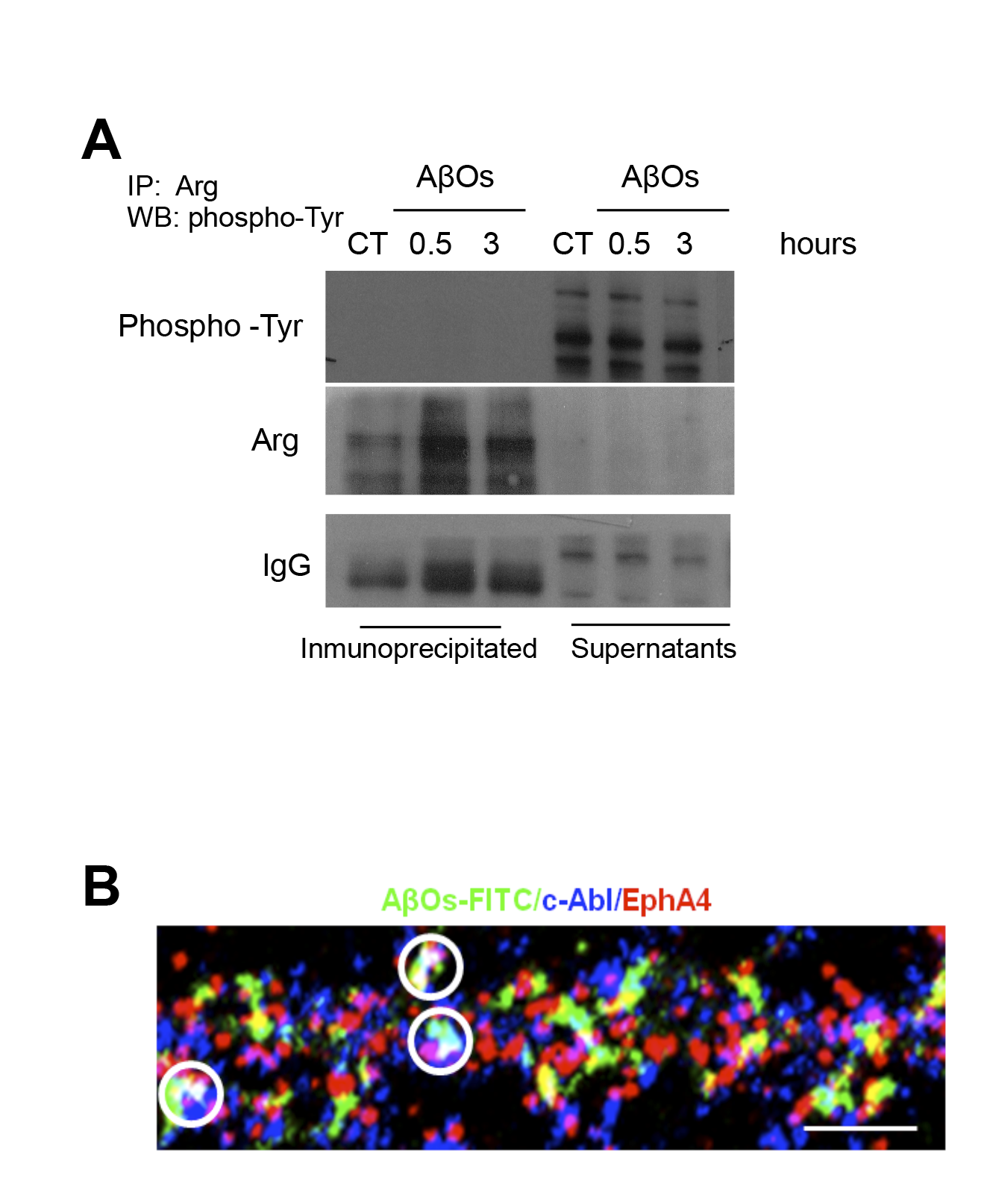

Supplement: Figure S2 — (A) Cultured hippocampal neurons (15 DIV) were treated with 3 μM AβOs for 0.5 and 3 hours. Arg was immunoprecipitated and then analyzed by immunoblotting with an anti-phosphotyrosine antibody. (image representative of three independent experiments). (B) Hippocampal neurons were treated for 90 minutes with AβOs-FITC (green) and immunolabeled for EphA4 (red) and c-Abl (blue). The circles show examples of co-localization of the 3 labels. Scale bar, 5 μm. (TIF) [file pone.0092309.s002.tif]

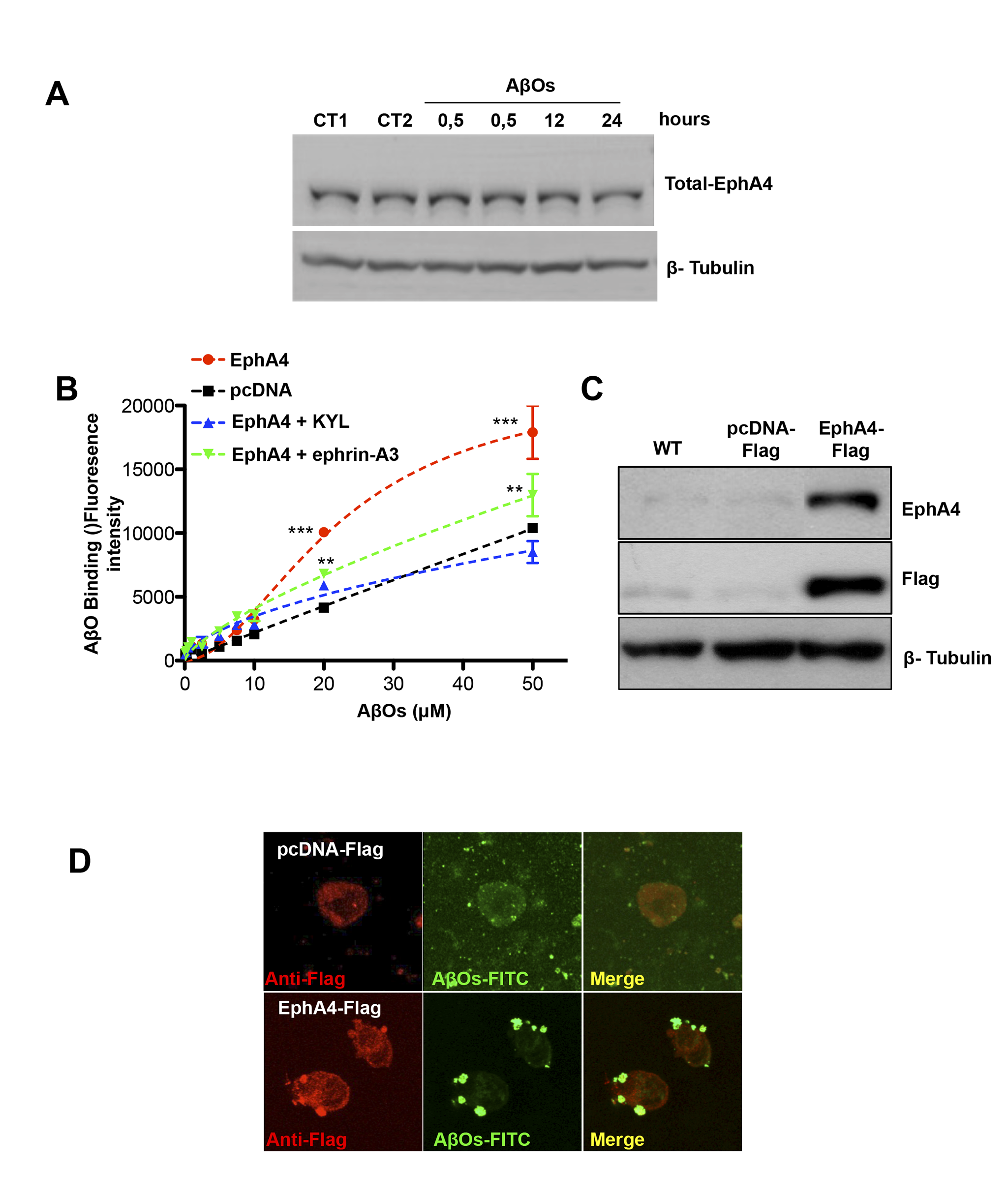

Supplement: Figure S3 — (A) Cultured hippocampal neurons (7 DIV) were treated with 5 μM AβOs for 0.5 to 24 hours. Immunoblot showing total EphA4 levels (B) HEK293 cells overexpressing EphA4-Flag show increase binding of AβOs-FITC compared to controls cells (empty vector), while pre-incubation with the specific inhibitor KYL or the ephrin-A3 ligand of EphA4 receptor displaced AβOs-FITC binding. The data was fitted to a one site-specific binding curve (dash line) obtaining a Kd of 22 μM for AβOs-FITC binding in cells overexpressing EphA4 receptor. Data was analized by two-anova followed by Bonferroni's test. (**p<0.01, *** p<0.001) (C). HEK293 cells that overexpress EphA4-Flag or pcDNA-Flag. Immunoblotting was performed to detect EphA4 and Flag. (D) Immunofluorescence labeling for the Flag epitope (red) and AβOs-FITC labeling (green) of pcDNA-Flag and EphA4-Flag expressed in HEK293 cells. Representative confocal microscopy images are shown. (TIF) [file pone.0092309.s003.tif]

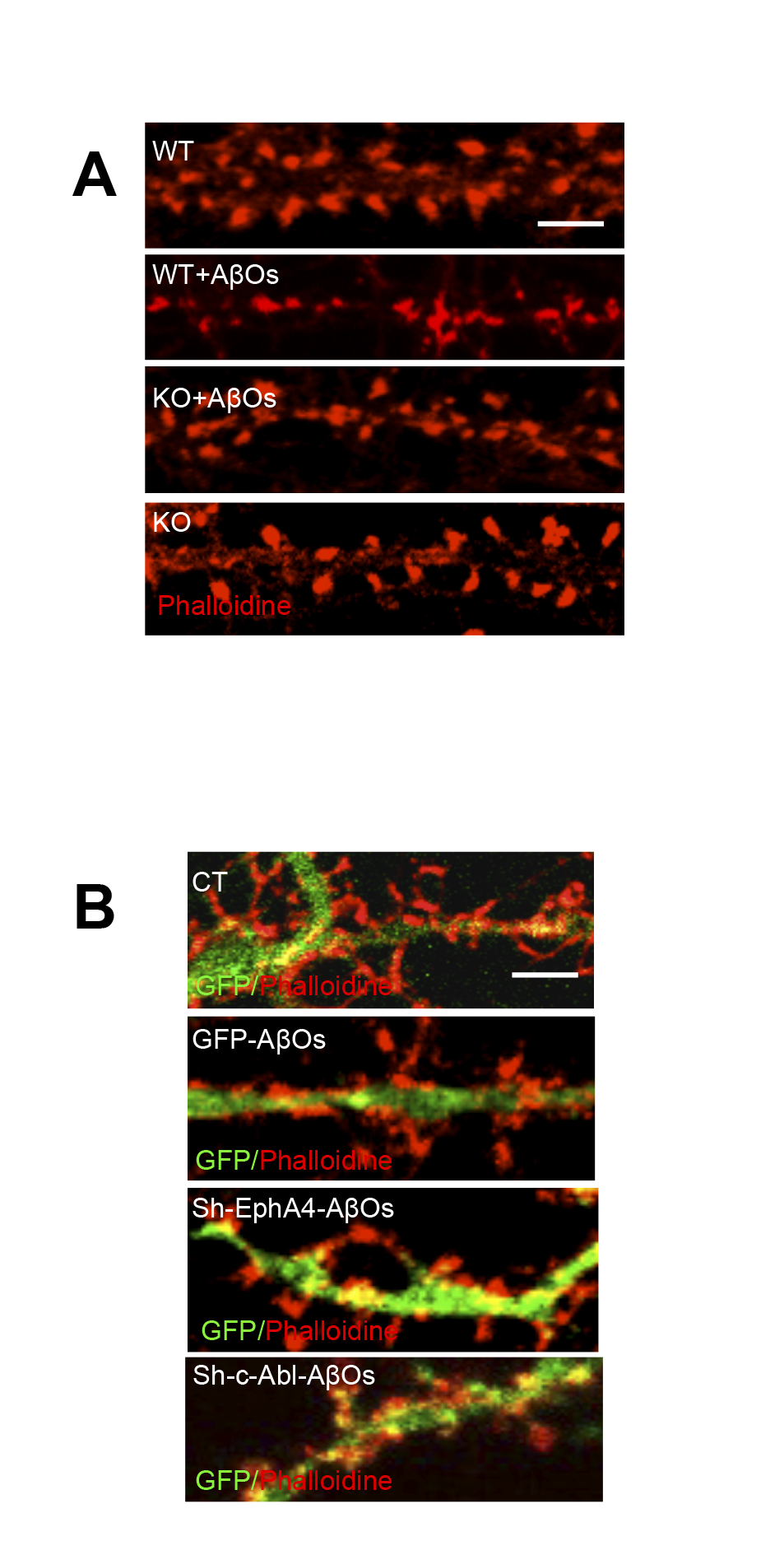

Supplement: Figure S4 — (A) Dendritic spines of wild-type (EphA4+/+, WT) or EphA4 knockout (EphA4-/-, KO) cultures of hippocampal neurons (15 DIV) exposed to AβOs for 5 hours. Confocal images showing phalloidin-TRITC staining (red). Scale bar, 5 μm. (B) Neurons transfected with pGFP, sh-EphA4, sh-c-Abl (green), scramble RNA EphA4 and c-Abl (SC) and treated with AβOs for 5 hours. Confocal images showing phalloidin-TRITC (red) and GFP (green) Scale bar, 5 μm. (TIF) [file pone.0092309.s004.tif]
